# The mechanism of abscisic acid regulation of wild *Fragaria* species in response to cold stress

**DOI:** 10.1186/s12864-022-08889-8

**Published:** 2022-09-26

**Authors:** Jincheng Shen, Jie Liu, Yongge Yuan, Luxi Chen, Junxia Ma, Xin Li, Junmin Li

**Affiliations:** 1 Zhejiang Provincial Key Laboratory of Plant Evolutionary Ecology and Conservation, Taizhou University, Taizhou, 318000 China; 2 College of Life Science and Medicine, Zhejiang Sci-Tech University, Hangzhou, 310018 China; 3 Department of Plant and Environmental Sciences, University of Copenhagen, 1871 Frederiksberg C, Denmark

**Keywords:** Low temperature stress, Wild strawberry, Abscisic acid, Glucose, Fructose

## Abstract

**Background:**

Abiotic stresses have increasingly serious effects on the growth and yield of crops. Cold stress, in particular, is an increasing problem. In this study, *Fragaria daltoniana* and *F. vesca* were determined to be cold-resistant and cold-sensitive species, respectively. Integrated transcriptomics and metabolomics methods were used to analyze the regulatory mechanism of abscisic acid (ABA) in *F. daltoniana* and *F. vesca* in their response to low temperature stress.

**Results:**

*F. daltoniana* and *F. vesca* increased their ABA content under low temperature stress by upregulating the expression of the ABA biosynthetic pathway gene *NCED* and downregulating the expression of the ABA degradative gene *CYP707A*. Both types of regulation increased the accumulation of glucose and fructose, resulting in a reduction of damage under low temperature stress. Twelve transcription factors were found to be involved in the ABA regulatory pathway. The strong cold tolerance of *F. daltoniana* could be owing to its higher levels of ABA that accumulated compared with those in *F. vesca* under low temperature stress. In addition, the gene *ABF2*, which is related to the transduction of glucose signaling, was significantly upregulated in the leaves of *F. daltoniana*, while it was downregulated in the leaves of *F. vesca* under low temperature stress. This could contribute to the higher levels of glucose signal transduction in *F. daltoniana*. Thus, this could explain the higher peroxidase activity and lower damage to cell membranes in the leaves of *F. daltoniana* compared with *F. vesca* under low temperature stress, which endows the former with stronger cold tolerance.

**Conclusions:**

Under low temperature stress, the differences in the accumulation of ABA and the expression trends of *ABF2* and *ABF4* in different species of wild strawberries may be the primary reason for their differences in cold tolerance. Our results provide an important empirical reference and technical support for breeding resistant cultivated strawberry plants.

**Supplementary Information:**

The online version contains supplementary material available at 10.1186/s12864-022-08889-8.

## Introduction

Low temperature is a typical abiotic stress factor, which can significantly affect plant growth, development, and crop yields [1]. Low temperature stress is the primary reason for the decline in the yield and quality of many crops in temperate and arid regions [2]. The global annual loss of agricultural productivity caused by low temperature stress is approximately 2 billion USD [3]. Moreover, many agronomically important crops cannot be improved by cold domestication, including rice, corn, soybeans, potatoes, cotton, and tomatoes [4]. Therefore, exploring the response and tolerance mechanism of plants under low temperature stress is highly important to improve the cold tolerance of plants and increase the quality and yield of crops.

Strawberry is highly nutritious and rich in vitamin C, anthocyanins, and other beneficial substances [5]. However, the resistance of cultivated strawberries is limited [6]. Improving the cold tolerance of strawberries is an important goal of strawberry breeding programs. As a species that is related to cultivated strawberry, wild strawberry can strongly resist stress and offers abundant germplasm resources for research on the mechanisms of resistance in the cultivated strawberry [7]. To accelerate the genetic improvement and breeding of cultivated strawberry, it is necessary to determine the responses of wild *Fragaria* species to low temperatures and the possible mechanisms they use to regulate them.

The cold stress response mechanisms of plants are very complex, and the signal pathways primarily include oxidation pathways, abscisic acid (ABA), mitogen-activated protein kinase, *Arabidopsis* response modifier, and Ca^2+^, among others [8]. ABA is a phytohormone that is found at low levels in plants but has substantial effects [9]. It plays an important role in the signal integration and regulation of the stress response genes in low temperature stress [9]. After binding to ABA receptors, ABA enhances the resistance of plants to stress by inducing changes in the expression of ABA-responsive genes, such as regulating the synthesis of osmotic adjustment substances that include proline, betaine and soluble sugar to reduce the loss of water from cells and maintain cell membrane stability [10, 11]; reducing the damage to plant photosynthesis from stress to ensure the synthesis of plant carbohydrates and energy metabolism [12]; upregulating the expression of antioxidant enzyme genes and enhancing the activity of antioxidant enzymes to reduce damage from the accumulation of reactive oxygen species (ROS) in the membrane system [13].

Genes that are involved in the cold response of strawberry leaves have been identified [14]. Chen et al. [14] found that environmental adaptation and carbohydrate metabolic pathways were significantly enriched in cultivated strawberries under low temperature stress and identified three CBF/DREB transcription factors and one cold-responsive (COR) gene that may play a role in cold resistance. In addition, various proteins and metabolites in strawberry have been evaluated [6, 15]. The cold tolerant mechanisms were also determined. For example, the exogenous application of ABA or glycine betaine has been verified to be effective in inducing cold tolerance in strawberry. Recently, an integrated analysis of physiological indicators, transcriptomics and metabolomics was used to explore the network that strawberry uses to respond to low temperature stress. The cold signaling initiated the expression of downstream COR genes with the *cis*-acting element ABRE or CRT/DRE in the ABA-independent or ABA-dependent pathways to induce plant defense against this stress. Notably, ABA also plays an important role in the cold tolerance of polyploidy *Fragaria* plants. An analysis of the transcriptomic changes in the autotetraploid strawberry *F. nilgerrensis* revealed that the coordination of Ca^2+^ signal transduction, transcription factors, ROS scavenging enzymes, phenylpropanoid biosynthesis, ABA signaling and carbohydrate metabolism play important roles in enhancing its resistance to cold stress. Notably, tetraploid *F. moupinensis* had higher concentrations of ABA and contents of soluble sugars and non-structural carbon (NSC), indicating that ABA has a regulatory role in the accumulation of soluble sugar and NSC in the responses of *F. moupinensis* to cold stress [16]. However, the mechanisms involved with the ABA regulatory pathway are still poorly understood.

Currently, research on the mechanisms of cold tolerance in strawberry mostly focuses on analyzing the responses observed through transcriptomic or metabolomic studies of plants under low temperature stress. In addition, the responses of cold-tolerant and cold-sensitive plant species differ [17], and the differences in the response mechanisms of strawberry species that differ in cold resistance are poorly understood. In this study, transcriptome, metabolome and physiological index data were integrated and analyzed to examine the regulatory mechanism of ABA in wild strawberry under low temperature stress. Our results provide an important empirical reference and key technical support for breeding resistant cultivated strawberry plants.

## Results

### Effect of low temperature stress on physiological indices

**Fig. 1:**
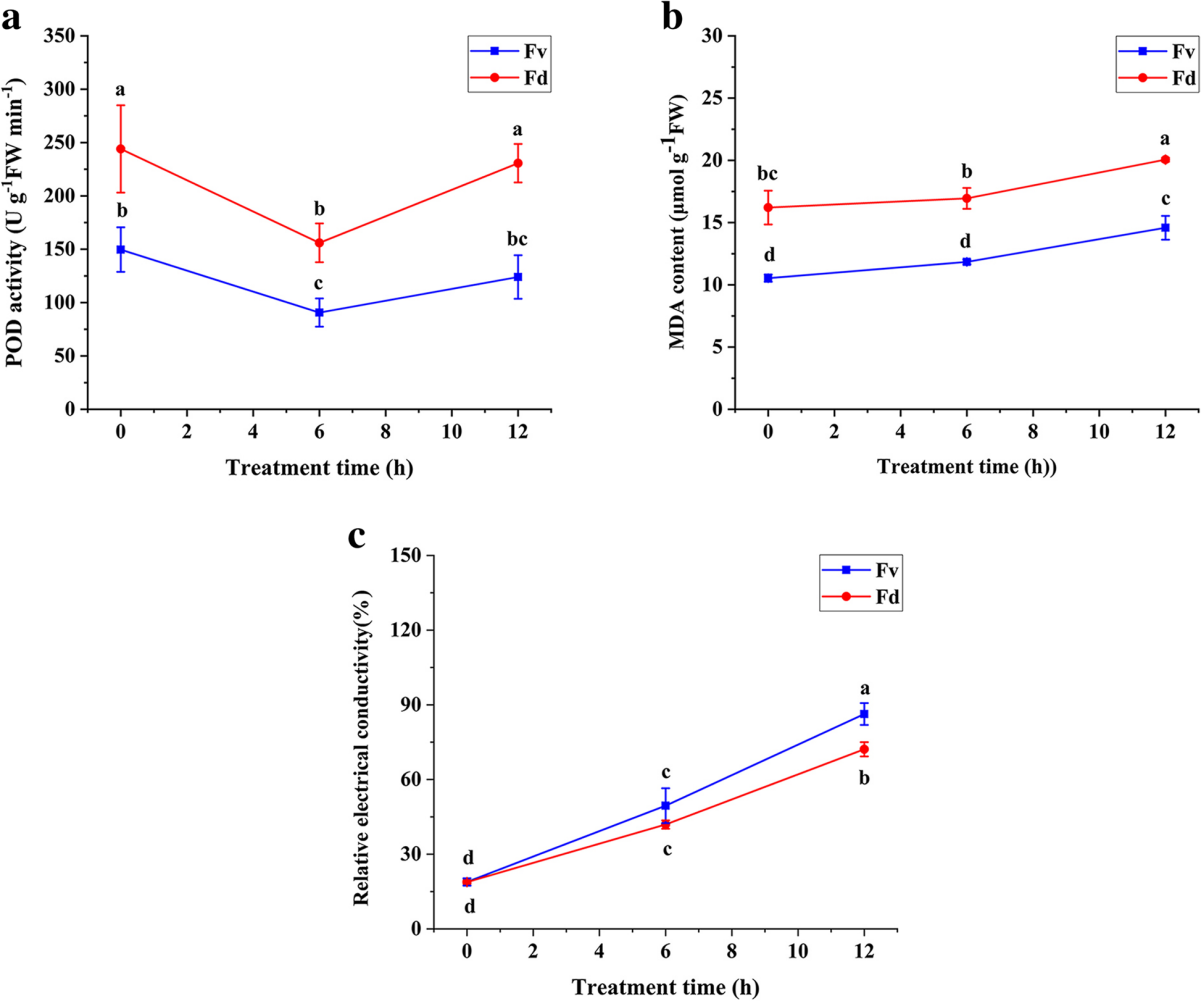
The changes in peroxidase (POD) activity (**a**), malondialdehyde (MDA) content (**b**), and relative electrical conductivity (**c**) of wild strawberry leaves under low temperature stress. Fv, *Fragaria vesca*; Fd, *F. daltoniana*. Different lowercase letters indicate significant differences between different treatments

Low temperature stress increased the malondialdehyde (MDA) content and relative electrical conductivity (REC) of the leaves of *F. daltoniana* and *F. vesca*, while it caused the peroxidase (POD) activity to first decrease and then increase (Fig. 1). Compared with the leaves of untreated *F. daltoniana* and *F. vesca*, the POD activity of leaves of *F. daltoniana* and *F. vesca* both decreased significantly under low temperature treatment for 6 h, and the REC increased significantly (Fig. 1). The MDA content and REC in the leaves of *F. daltoniana* and *F. vesca* both increased significantly, and the POD activity of the leaves of *F. daltoniana* increased significantly under low temperature treatment for 12 h (Fig. 1).

### Effect of low temperature stress on ABA accumulation and signal transduction

**Fig. 2:**
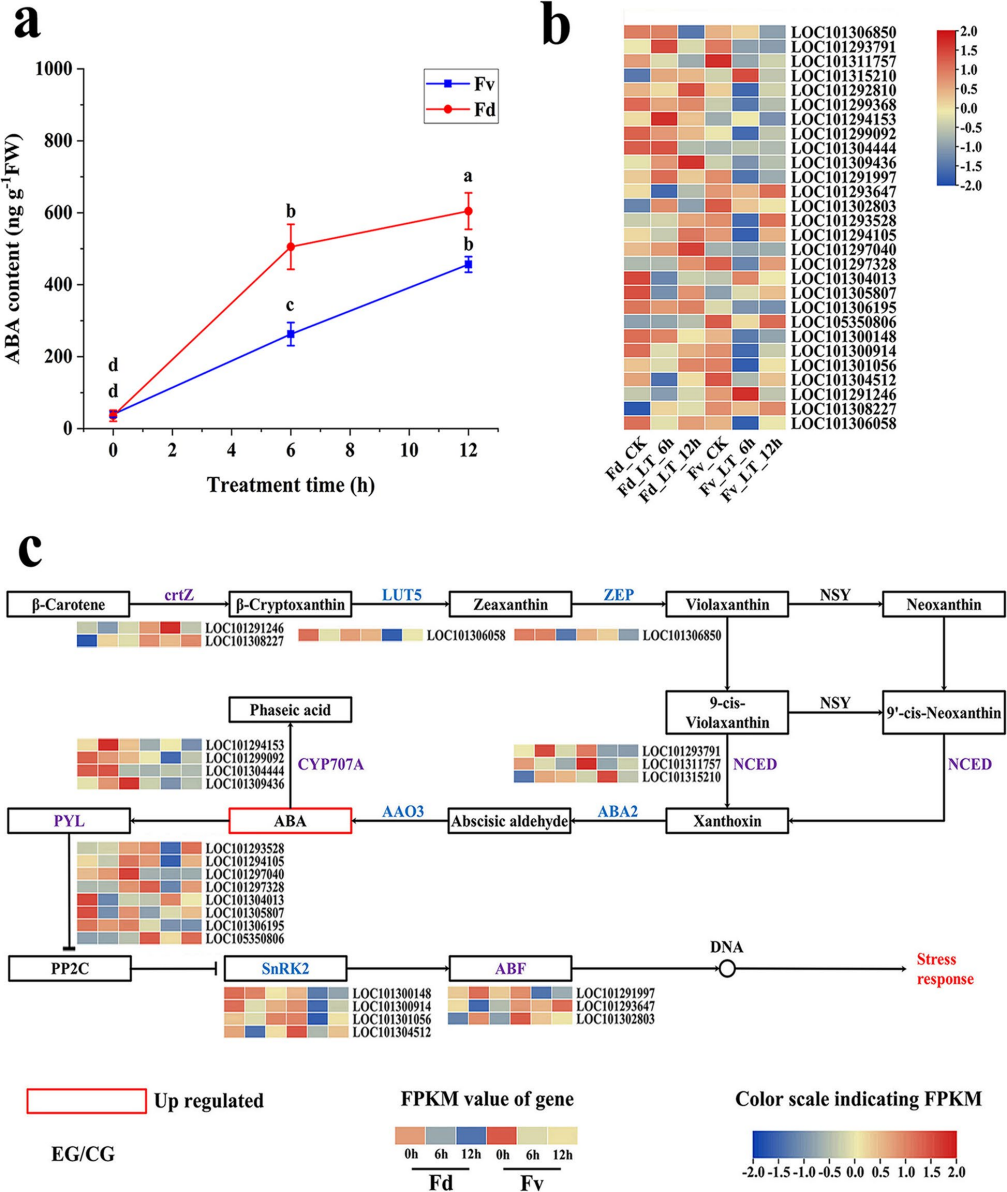
The cold tolerance mechanism of *Fragaria vesca* and *F. daltoniana*. **a** The changes in ABA content in the leaves of wild strawberries under low temperature stress. **b** A heat map of genes related to the ABA pathway. **c** Synthesis, degradation and response pathways of ABA. Fv, *F. vesca*; Fd, *F. daltoniana*; CK, control group; LT, low temperature treatment; EG, experimental group; ABA, abscisic acid; FPKM, fragments per kilobase of transcript per million mapped fragments. Different lowercase letters indicate the significant differences between different treatments

Compared with the leaves of untreated *F. daltoniana* and *F. vesca*, the ABA content of leaves of *F. daltoniana* and *F. vesca* both increased significantly under 6 h and 12 h of low temperature treatment (Fig. 2a). A total of 28 DEGs were found to be involved in the biosynthesis of ABA and the degradation and signal transduction pathways of *F. daltoniana* and *F. vesca* under low temperature stress (Fig. 2b). Compared with the leaves of untreated *F. daltoniana* and *F. vesca*, the ABA biosynthetic pathway gene *NCED* (LOC101315210) in the *F. daltoniana* and *F. vesca* leaves was significantly upregulated, and the ABA degradative gene *CYP707A* (LOC101299092) was significantly downregulated under low temperature stress (Fig. 2c). In addition, three genes that may play a role in the accumulation of ABA in wild strawberry leaves under low temperature stress were also detected, including *crtZ* (LOC101308227), *NCED* (LOC101293791), and *CYP707A* (LOC101294153). Compared with the leaves of untreated *F. daltoniana* and *F. vesca*, the expression of *crtZ* (LOC101308227) in the leaves of *F. daltoniana* was significantly upregulated under low temperature stress. The expression of *NCED* (LOC101293791) and *CYP707A* (LOC101294153) changed in different manners in the leaves of two wild strawberry species (Fig. 2c), indicating variation in the regulatory mechanisms of ABA biosynthesis between the two species. Compared with the leaves of untreated *F. daltoniana* and *F. vesca*, the expression level of *ABF4* (LOC101291997) in the *F. vesca* leaves was significantly downregulated, while that of *bZIP*3 (LOC101293647) was significantly downregulated in *F. daltoniana*. *ABF2* (LOC101302803) was significantly upregulated in *F. daltoniana* and downregulated in *F. vesca* under low temperature stress (Fig. 2c).

### Weighted gene co-expression network analysis

**Fig. 3:**
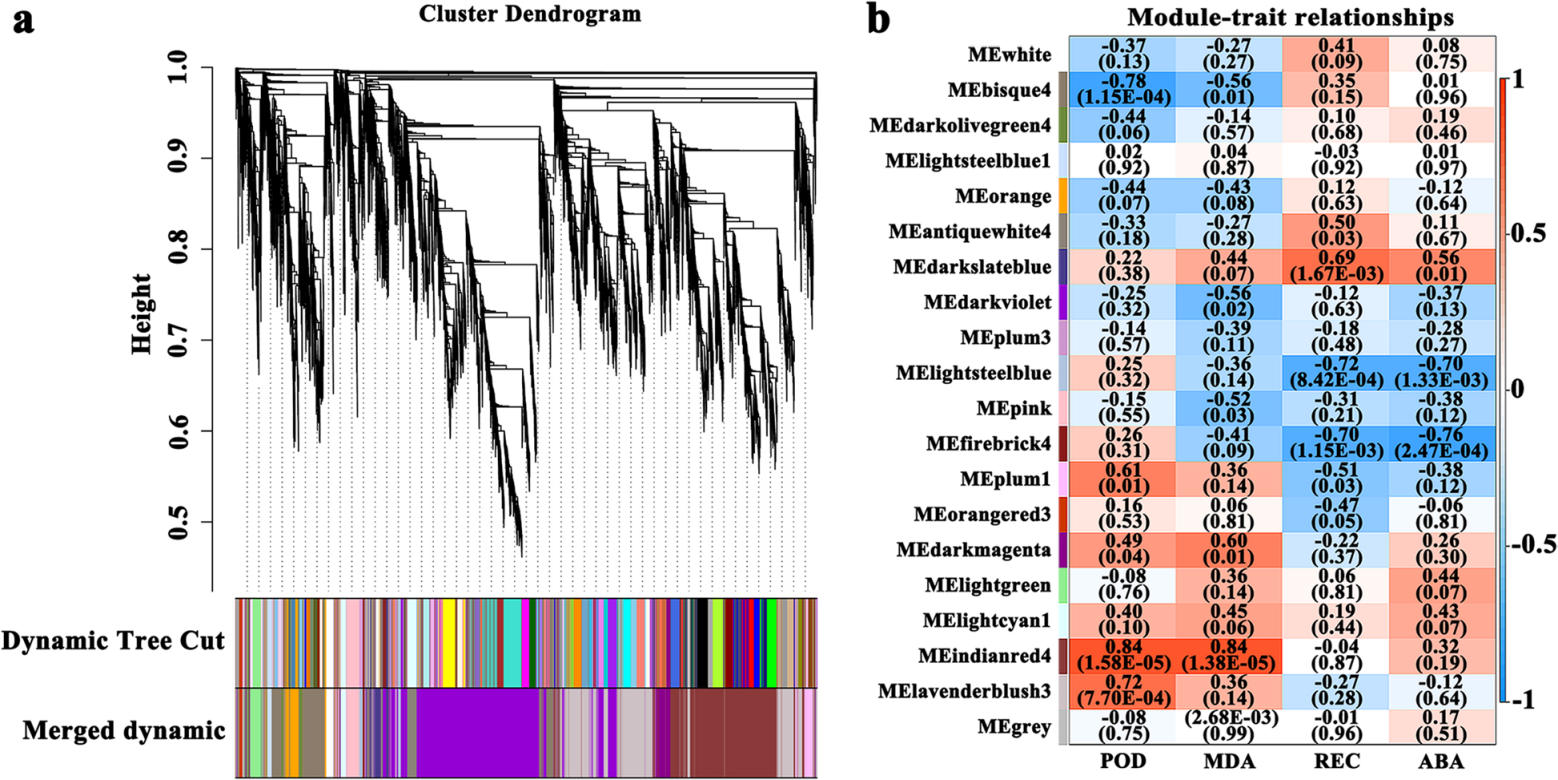
Weighted gene co-expression network analysis of physiological traits and mRNAs. **a** Cluster dendrogram. **b** Correlation diagram characterizing the relationships between the modules and physiological indicators. ABA, abscisic acid; MDA, malondialdehyde; POD, peroxidase; REC, relative electrical conductivity

To clarify the cold tolerance mechanism of wild strawberries, we combined the transcriptome and physiological indicators to construct a gene co-expression network of 18 strawberry samples under low temperature stress. After filtering out poorly expressed genes (fragments per kilobase of transcript per million mapped fragments [FPKM] < 0.5), the soft threshold was set to 27 by calculating the adjacency function between genes (Fig. S1). Twenty modules were constructed using the weighted gene co-expression network analysis (WGCNA) algorithm. In Fig. 3, the darkslateblue module is closely related to the REC and ABA content, and the indianred4 module is closely related to the POD activity and MDA content.

### GO and KEGG function annotation and enrichment analyses

The Gene Ontology (GO) and Kyoto Encyclopedia of Genes and Genomes (KEGG) function annotation and enrichment analyses of the differentially expressed genes (DEGs) between the samples treated at low temperatures and the control samples and gene modules closely related to plant physiological indicators revealed that the “glycine, serine and threonine metabolic pathway” and “starch and sucrose metabolic pathway” may play important roles in the regulation of ABA in response to low temperature stress in wild strawberries (Figs. S2–S4).

### Combined analysis of transcription and metabolism

**Fig. 4:**
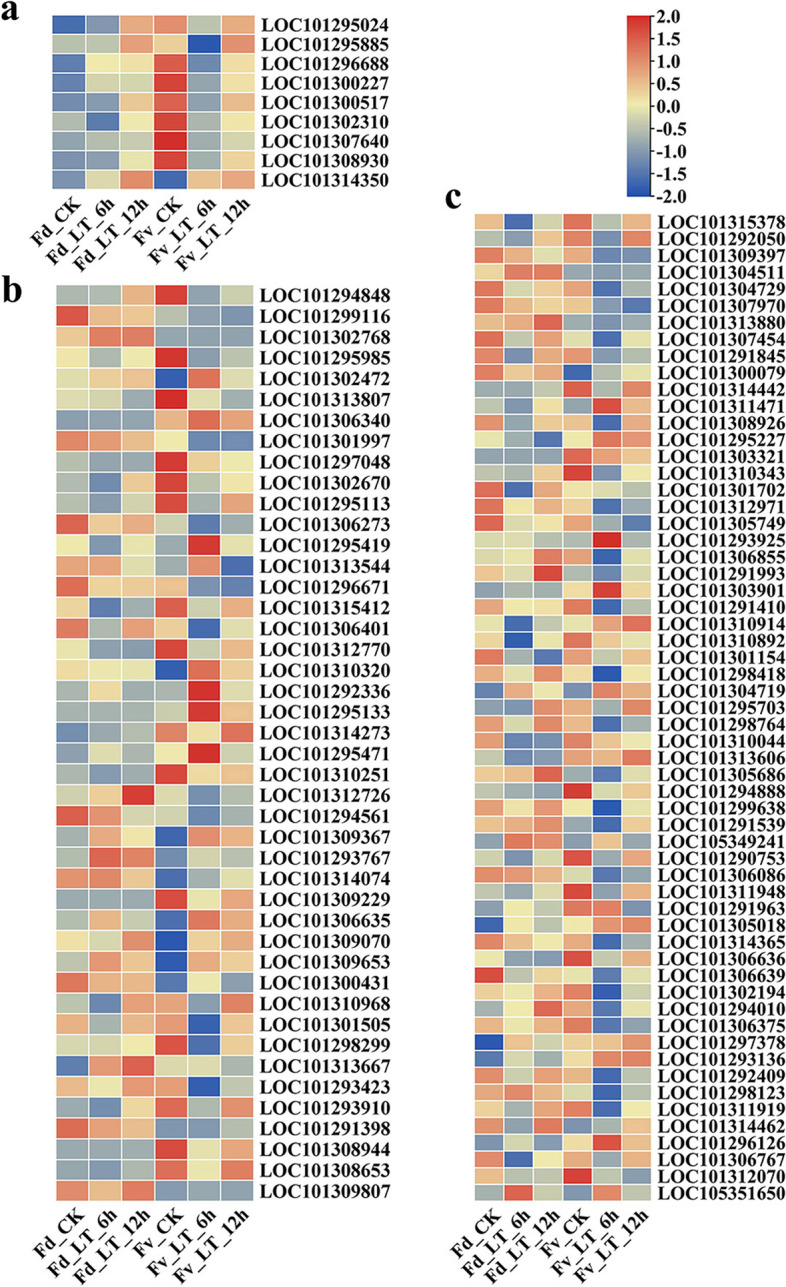
The heat maps of differentially expressed genes and transcription factors. **a** The heat map of differentially expressed transcription factors in ABA content-related gene modules. **b** The DEGs in starch and sucrose metabolic pathways. **c** The DEGs in amino acid metabolic pathway. Fv, *F. vesca*; Fd, *F. daltoniana*; CK, control group; LT, low temperature treatment; DEGs, differentially expressed genes; ABA, abscisic acid

**Fig. 5:**
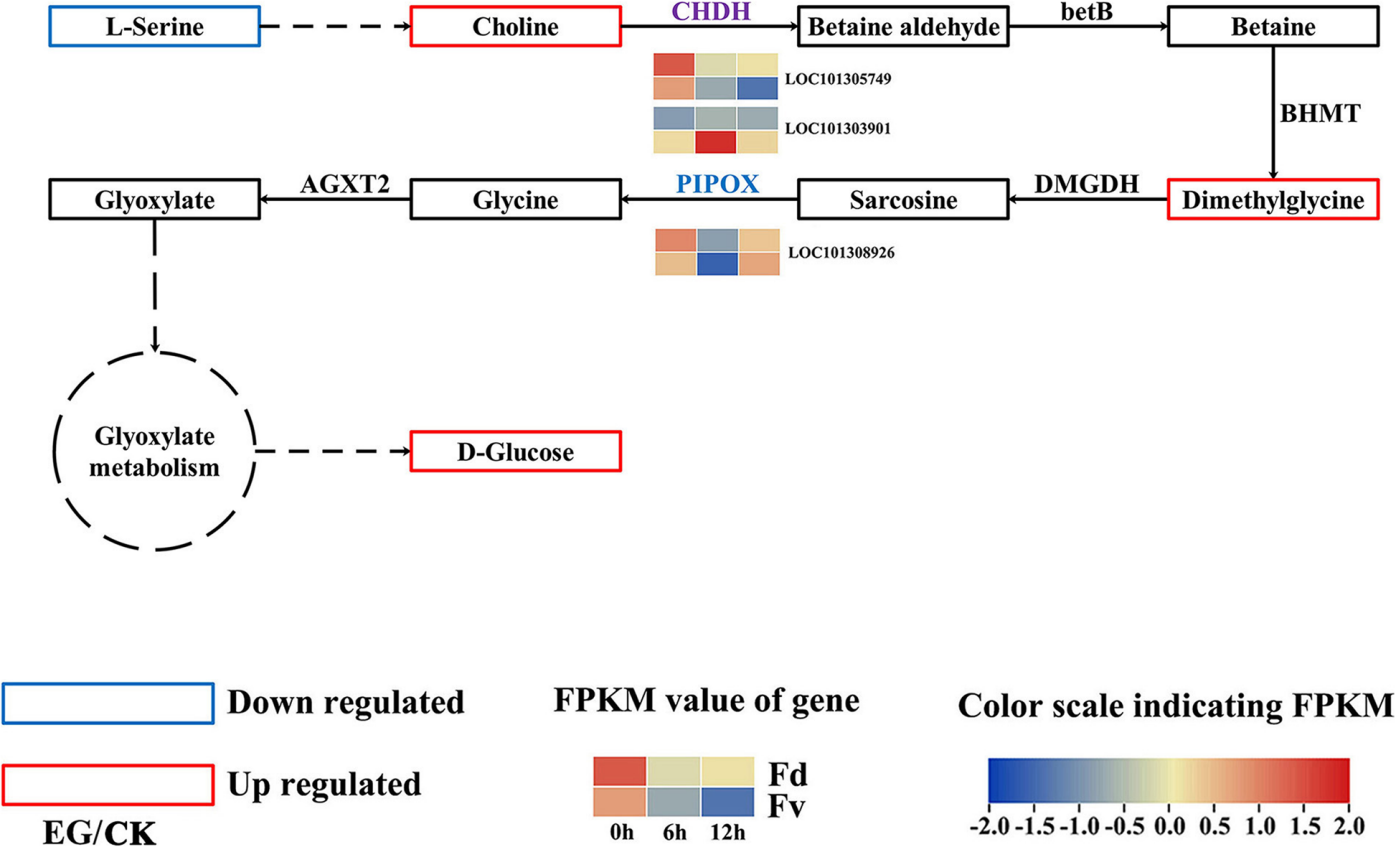
Metabolic pathway diagram of wild strawberry glycine, serine, and threonine. Fv, *F. vesca*; Fd, *F. daltoniana*; EG, experimental group; CK, control group; CHDH, choline dehydrogenase; PIPOX, sarcosine oxidase / l-pipecolate oxidase

**Fig. 6:**
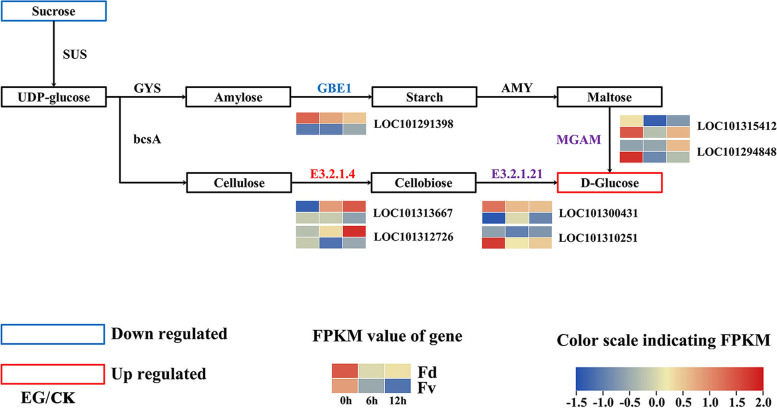
Metabolic pathway diagram of wild strawberry starch and sucrose. Fv, *F. vesca*; Fd, *F. daltoniana*; EG, experimental group; CK, control group; GBE1, 1,4-α-glucan branching enzyme; E3.2.1.4, *endo 1,4-β-d-glucanase*; E3.2.1.21, ß-glucosidase; MGAM, maltase-glucoamylase

After low temperature stress, the content of almost all the differentially accumulated amino acids in the leaves of *F. daltoniana* and *F. vesca* decreased significantly compared with the untreated leaves, indicating that the rate of consumption of amino acids in strawberry leaves was higher than that under low temperature stress. An analysis of amino acid metabolic pathway genes found that “tryptophan metabolism,” “arginine and proline metabolism,” “glycine, serine and threonine metabolism,” “tyrosine metabolism,” and “valine, leucine and isoleucine degradation” were differentially expressed. A total of 19 DEGs were significantly upregulated, including LOC101304511, LOC101313880, LOC101300079, LOC101311471, LOC101295227, LOC101293925, LOC101306855, LOC101291993, LOC101303901, LOC101304719, LOC101305686, LOC105349241, LOC101305018, LOC101294010, LOC101297378, LOC101293136, LOC101314462, LOC101296126, and LOC105351650 (Fig. 4a). These genes may play roles in amino acid metabolism. Choline dehydrogenase (CHDH) can catalyze choline, an intermediate product of glycine, serine, and threonine metabolism, to provide a substrate for the synthesis of dimethylglycine (Fig. 5). Compared with the leaves of untreated *F. daltoniana* and *F. vesca*, *CHDH* (LOC101303901) increased 3.77-, 3.31-, 2.45-, and 1.09-fold under 6 and 12 h of low temperature stress in *F. daltoniana* and *F. vesca*, respectively, and the content of dimethylglycine increased 54.65-, 53.53-, 7.87-, and 7.28-fold, respectively (Fig. 6, Table 1, and Table S3). Therefore, *CHDH* (LOC101303901) may play a key role in the amino acid metabolic pathway of wild strawberry leaves under low temperature stress.

**Table 1:** The content of amino acids and intermediate metabolites in their metabolic pathways in leaves of *F. vesca* and *F. daltoniana* under cold stress

Metabolite	Fd_CK	Fd_LT_6h	Fd_LT_12h	Fv_CK	Fv_LT_6h	Fv_LT_12h
L-Arginine	3.75E+ 06	1.59E+ 06	1.66E+ 06	3.89E+ 05	3.94E+ 05	4.46E+ 05
L-Asparagine	1.64E+ 07	8.10E+ 06	6.68E+ 06	3.90E+ 05	3.98E+ 05	3.02E+ 05
L-Aspartic Acid	8.49E+ 06	2.38E+ 06	1.62E+ 06	2.10E+ 06	1.02E+ 06	7.64E+ 05
L-Citrulline	1.12E+ 05	3.64E+ 04	5.01E+ 04	2.03E+ 04	3.96E+ 04	1.40E+ 04
L-Cysteine	6.08E+ 04	5.91E+ 04	4.20E+ 04	5.51E+ 04	5.14E+ 04	4.38E+ 04
L-Cystine	1.46E+ 04	5.23E+ 04	3.97E+ 04	8.47E+ 03	3.05E+ 04	2.55E+ 04
L-Glutamic acid	5.90E+ 07	1.52E+ 07	8.20E+ 06	3.04E+ 07	1.63E+ 07	1.24E+ 07
L-Histidine	1.04E+ 05	7.77E+ 04	6.13E+ 04	3.36E+ 04	3.35E+ 04	4.03E+ 04
L-Homocitrulline	1.70E+ 04	1.89E+ 04	2.35E+ 04	2.30E+ 04	1.96E+ 04	3.34E+ 04
L-Homocystine	1.89E+ 04	2.53E+ 04	2.84E+ 04	2.01E+ 04	2.71E+ 04	3.56E+ 04
L-Homomethionine	1.37E+ 05	5.99E+ 04	5.13E+ 04	6.44E+ 04	5.55E+ 04	4.75E+ 04
L-Isoleucine	4.46E+ 06	5.50E+ 06	5.75E+ 06	5.57E+ 06	5.01E+ 06	4.85E+ 06
L-Leucine	4.24E+ 06	5.31E+ 06	5.72E+ 06	5.38E+ 06	4.61E+ 06	4.61E+ 06
L-Lysine	1.31E+ 06	1.09E+ 06	1.11E+ 06	9.64E+ 05	1.12E+ 06	1.05E+ 06
L-Methionine	3.57E+ 05	6.18E+ 05	6.73E+ 05	3.40E+ 05	3.79E+ 05	3.54E+ 05
L-Norleucine	4.30E+ 06	5.21E+ 06	5.90E+ 06	5.53E+ 06	5.05E+ 06	4.89E+ 06
L-Ornithine	9.99E+ 05	5.04E+ 05	4.48E+ 05	2.21E+ 04	2.90E+ 04	2.16E+ 04
L-Phenylalanine	8.43E+ 06	1.02E+ 07	1.21E+ 07	5.10E+ 06	6.38E+ 06	6.83E+ 06
L-Proline	1.68E+ 07	1.65E+ 07	1.75E+ 07	1.52E+ 07	2.19E+ 07	1.57E+ 07
L-Serine	1.80E+ 06	7.16E+ 05	1.11E+ 06	1.70E+ 05	1.60E+ 05	1.26E+ 05
L-Threonine	4.31E+ 06	2.70E+ 06	2.21E+ 06	2.23E+ 06	1.73E+ 06	1.71E+ 06
L-Tryptophan	2.89E+ 06	3.22E+ 06	3.18E+ 06	2.23E+ 06	2.82E+ 06	4.37E+ 06
L-Tyrosine	8.62E+ 06	6.74E+ 06	8.97E+ 06	8.08E+ 06	1.30E+ 07	1.39E+ 07
L-Valine	3.86E+ 07	3.86E+ 07	3.68E+ 07	4.25E+ 07	4.45E+ 07	3.54E+ 07
Dimethylglycine	2.46E+ 05	1.34E+ 07	1.31E+ 07	1.20E+ 06	9.45E+ 06	8.74E+ 06

Compared with the leaves of untreated *F. daltoniana*, 22 significantly upregulated genes were detected in the “starch and sucrose metabolism” pathway of *F. daltoniana* under low temperature stress, including LOC101294848, LOC101302768, LOC101302472, LOC101306340, LOC101295419, LOC101313544, LOC101310320, LOC101292336, LOC101295133, LOC101314273, LOC101295471, LOC101312726, LOC101309367, LOC101293767, LOC101314074, LOC101306635, LOC101309070, LOC101309653, LOC101300431, LOC101310968, LOC101313667, and LOC101293910 (Fig. 4b). These genes may play roles in the breakdown of sucrose into fructose and glucose. Endoglucanase can catalyze cellulose, the intermediate metabolite of the sucrose metabolic pathway, to provide a substrate for glucose synthesis (Fig. 6). Compared with the leaves of untreated *F. daltoniana*, *endo 1,4-β-d-glucanase* (*EGase*, LOC101313667) was upregulated 6.17- and 8.79-fold in the leaves of *F. daltoniana* under 6 and 12 h of low temperature stress, respectively; the glucose content increased 3.63- and 3.83-fold, respectively, and the sucrose content decreased 2.06- and 2.48-fold, respectively (Figs. 4b and 6, Tables 2 and S4). Therefore, the differential expression of *EGase* (LOC101313667) under low temperature stress is likely to affect the ratio of sucrose to glucose in leaves.

**Table 2:** The sugar content of leaves of *F. vesca* and *F. daltoniana* under cold stress

Metabolite	Fd_CK	Fd_LT_6h	Fd_LT_12h	Fv_CK	Fv_LT_6h	Fv_LT_12h
D-(−)-Threose	1.80E+ 06	1.07E+ 06	7.14E+ 05	4.78E+ 04	7.50E+ 04	7.34E+ 04
D-Arabinose	4.88E+ 04	6.70E+ 04	9.72E+ 04	5.02E+ 04	8.39E+ 04	1.33E+ 05
D-Fructose	1.50E+ 06	5.83E+ 06	6.10E+ 06	2.14E+ 06	6.07E+ 06	9.13E+ 06
D-Glucose	1.59E+ 06	5.76E+ 06	6.08E+ 06	2.11E+ 06	6.05E+ 06	8.88E+ 06
D-Lactulose	8.42E+ 05	3.88E+ 05	4.18E+ 05	1.09E+ 06	7.77E+ 05	1.12E+ 06
D-Maltose	1.28E+ 06	8.74E+ 05	6.86E+ 05	2.44E+ 06	2.33E+ 06	3.15E+ 06
D-Panose	5.45E+ 03	3.67E+ 03	9.00E+ 00	2.66E+ 04	2.30E+ 04	3.66E+ 04
D-Ribose	1.46E+ 05	2.01E+ 05	1.78E+ 05	8.44E+ 04	1.46E+ 05	1.59E+ 05
D-Sucrose	3.09E+ 06	1.50E+ 06	1.25E+ 06	4.01E+ 06	3.10E+ 06	3.99E+ 06
D-Trehalose	7.88E+ 05	5.41E+ 05	6.08E+ 05	1.28E+ 06	1.13E+ 06	1.41E+ 06
Isomaltulose	1.62E+ 06	9.10E+ 05	9.57E+ 05	2.49E+ 06	2.20E+ 06	2.65E+ 06
Lactobiose	1.36E+ 06	7.65E+ 05	8.53E+ 05	2.35E+ 06	2.19E+ 06	2.55E+ 06
Melibiose	9.72E+ 05	5.44E+ 05	5.73E+ 05	1.36E+ 06	1.27E+ 06	1.51E+ 06
Raffinose	3.39E+ 04	1.60E+ 04	1.68E+ 04	1.18E+ 05	6.92E+ 04	1.13E+ 05
Sedoheptulose	6.58E+ 05	7.12E+ 05	6.91E+ 05	2.44E+ 05	3.02E+ 05	3.95E+ 05
Turanose	4.60E+ 06	2.95E+ 06	3.20E+ 06	2.86E+ 06	3.06E+ 06	2.19E+ 06

### Transcription factor prediction of the modular genes

The transcription factors were predicted based on the genes in the darkslateblue module that were closely related to the REC and ABA content, and nine differentially expressed transcription factors were identified (Fig. 4c). Each of the seven B3, bZIP, ERF, G2-like, GRAS, LBD, and MYB transcription factor families has one, and the NF-YB transcription factor family has two (Table 3).

**Table 3:** Family classification of differentially expressed transcription factors in abscisic acid (ABA) content-related gene modules

Gene ID	Transcription factor family	Transcription factor
LOC101300517	B3	MJB21.7
LOC101307640	bZIP	GBF1
LOC101314350	ERF	ERF9
LOC101295024	G2-like	PCL1
LOC101302310	GRAS	RGL1
LOC101300227	LBD	LBD1
LOC101296688	MYB	TT2
LOC101295885	NF-YB	NF-YB2
LOC101308930	NF-YB	NF-YB11

### Relation analysis between transcription factors and Core module genes

**Fig. 7:**
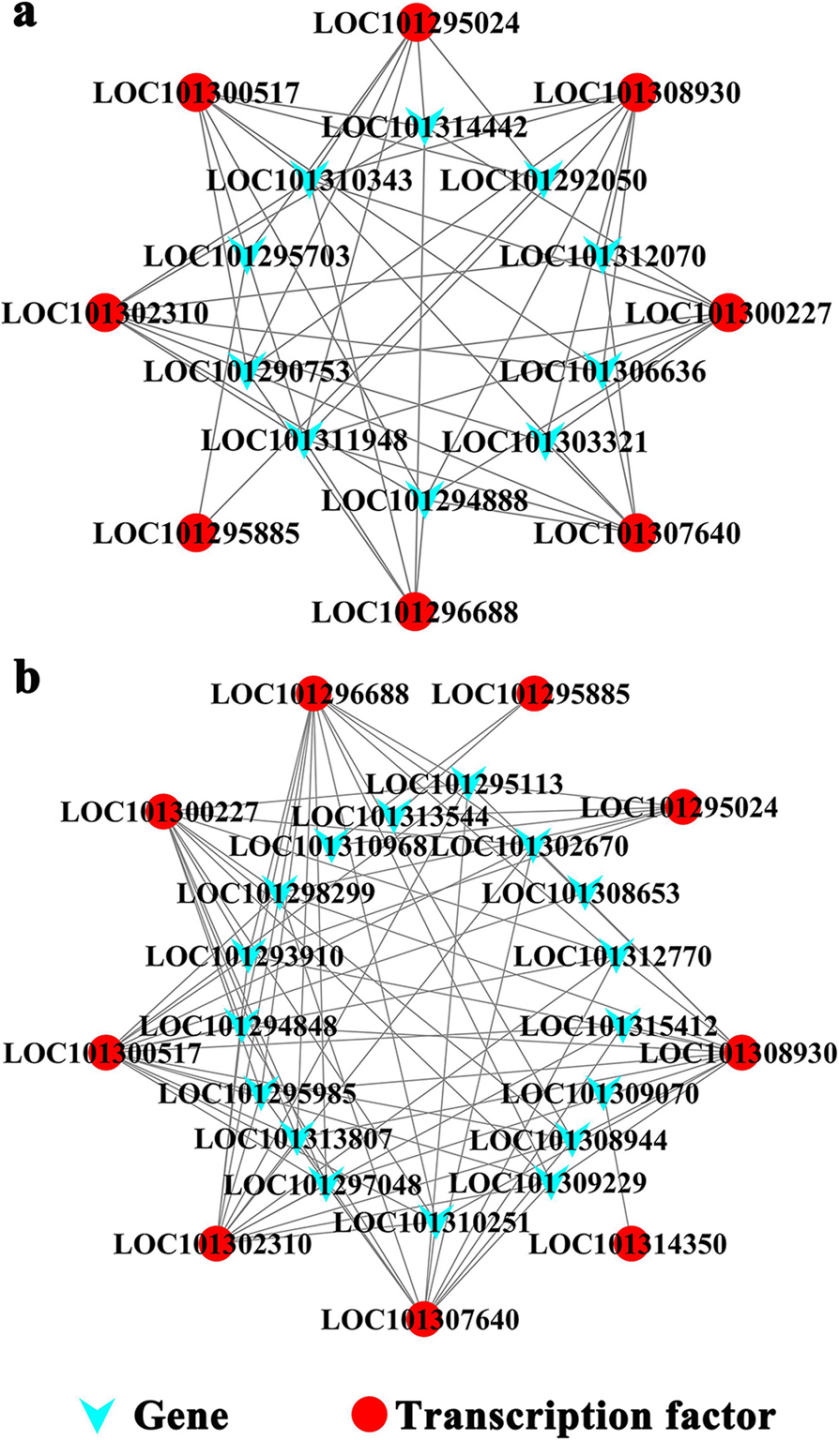
Network map of the differentially expressed transcription factors and genes. **a** Network map of the differentially expressed transcription factors and genes of the amino acid metabolic pathway. **b** Network map of the differentially expressed transcription factors and genes of the starch and sucrose metabolic pathways

A correlation analysis of differentially expressed transcription factors in the ABA module and the DEGs of starch and sucrose metabolic pathways and amino acid metabolic pathways was performed. The eight differentially expressed transcription factors were significantly correlated with the DEGs of 10 amino acid metabolic pathways (*P* < 0.05, *R* > 0.8). Nine differentially expressed transcription factors were significantly correlated with the DEGs of 17 starch and sucrose metabolic pathways (*P* < 0.05, *R* > 0.8) (Fig. 7).

### qRT-PCR validation

**Fig. 8:**
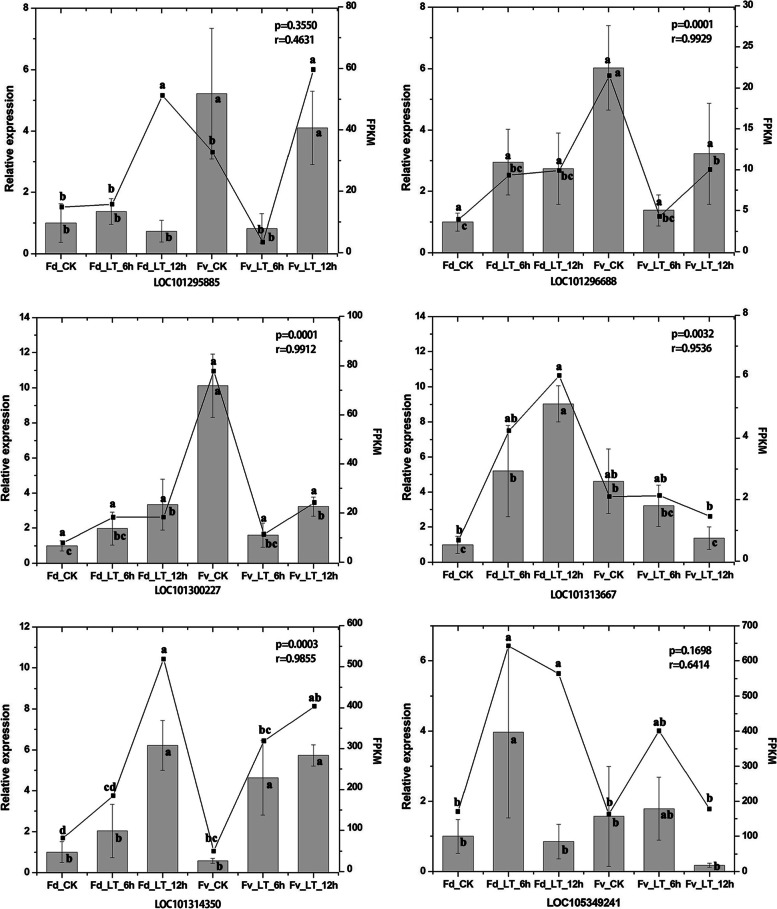
qRT-PCR validated the differentially expressed genes of the leaves of wild strawberries under low temperature stress. The bar graphs show the relative expression as measured by qRT-PCR, and the broken line graphs are the gene expression estimates obtained by RNA-Seq. Fv, *Fragaria vesca*; Fd, *F. daltoniana*; CK, control group; LT, low temperature treatment. qRT-PCR, real-time fluorescent quantitative reverse transcription PCR. Different lowercase letters indicate significant differences between different treatments

To verify the accuracy of the transcriptome sequencing results, we selected six DEGs that responded to low temperature stress for qRT-PCR validation. As shown in Fig. 8, the trends of expression of the six genes are basically consistent with the transcriptome sequencing results, which demonstrates that the transcriptome sequencing results are highly reliable and can be used to examine the cold tolerance mechanism of wild strawberries.

## Discussion

### Physiological response of wild strawberry under low temperature stress

The contents of REC and MDA in the leaves of *F. daltoniana* and *F. vesca* increased significantly under low temperature stress, while the POD activity first decreased significantly and then increased. These patterns could be owing to a reduction in the activity of ROS scavenging enzymes in response to the low temperature stress. An imbalance between ROS generation and scavenging leads to a large accumulation of ROS and membrane lipid peroxidation to synthesize MDA, which causes damage to the cell components and cell membranes, resulting in a significant increase in REC [18]. Previous studies have shown that the accumulation of ABA is essential for cold tolerance in plants. ABA can increase the content of osmotic adjustment substances in plants and the activity of ROS scavenging enzymes by regulating the expression of stress-responsive genes, thereby improving the cold tolerance of plants [19, 20]. The ABA contents in the leaves of the untreated *F. daltoniana* and *F. vesca* were very similar. After low temperature stress, the ABA content in the leaves of the two wild strawberries increased significantly, and the ABA content in the leaves of *F. daltoniana* (strong cold tolerance) was significantly higher than that of *F. vesca* (weak cold tolerance). After low temperature stress, the POD activity of the leaves of *F. daltoniana* was higher than that of *F. vesca*, and the REC was lower than that of *F. vesca*. Therefore, the accumulation of ABA in wild strawberry leaves under low temperature stress may affect its POD activity, improve the scavenging ability of ROS, reduce the oxidative damage caused by ROS, and protect the stability of the cell membrane structure.

### Sugar accumulation pathway of wild strawberry under low temperature stress

Plants often produce protective compounds, such as amino acids and soluble carbohydrates, in response to low temperature stress to reduce cell damage and preserve cell metabolism [16]. Previous studies showed that cold-tolerant plants accumulate more soluble carbohydrates and amino acids under low temperature stress than cold-sensitive plants [21, 22]. For example, Niu et al. [23] found that 37 amino acids and 33 sugars were accumulated in *Momordica charantia* seedlings with higher cold tolerance under low temperature, at higher levels than in seedlings with lower cold tolerance. However, in this study, the metabolomics data showed that the amino acid content in the leaves of *F. daltoniana* and *F. vesca* under lower temperature stress was significantly lower than those under normal temperature. The glucose and fructose contents increased significantly, and the sucrose content in the leaves of *F. daltoniana* decreased significantly. The sucrose content of *F. vesca* leaves decreased first and then increased to the level of the control group. The products of the carbohydrate and ketogenic amino acid metabolic pathways can be catalyzed to synthesize carbohydrates by a series of enzymes [24]. Therefore, the increase in glucose and fructose content is likely to be related to the metabolism of amino acids and sucrose. The amino acid content is affected by the genes of amino acid biosynthetic and metabolic pathways, while the carbohydrate content is affected by photosynthesis, sucrose and amino acid metabolism, and other gluconeogenesis pathways [24, 25]. Therefore, the upregulated genes involved in glycogenic amino acid metabolic, sugar-producing and ketogenic amino acid metabolic, and sucrose metabolic pathways in wild strawberry leaves may play important roles in changes in carbohydrate content under low temperature stress.

Choline dehydrogenase (CHDH) is an important enzyme in the glycine, serine, and threonine metabolic pathway (map 00260), which can catalyze choline, the intermediate product of this metabolic pathway to betaine aldehyde [26]. Betaine aldehyde is catalyzed by betaine aldehyde dehydrogenase, choline oxidase, and betaine homocysteine ​​methyltransferase to synthesize dimethylglycine, which then enters the glyoxylic acid metabolic pathway under the catalysis of a series of enzymes to provide sufficient substrates for glucose synthesis. Compared with the leaves of untreated *F. daltoniana* and *F. vesca*, the levels of expression of *CHDH* (LOC101303901) in *F. daltoniana* and *F. vesca* leaves after 6 and 12 h of low temperature stress were upregulated 3.77-, 3.31-, 2.45-, and 1.09-fold, respectively; the glycine content increased 54.65-, 53.53-, 7.87-, and 7.28-fold, respectively; the glucose content increased 3.63-, 3.83-, 2.86-, and 4.20-fold, respectively, and the fructose content increased 3.88-, 4.07-, 2.84-, and 4.28-fold, respectively. This indicates that *CHDH* is likely to play a key role in the amino acid metabolism and carbohydrate biosynthesis of wild strawberry under low temperature stress. Previous research has shown that the overexpression of *CHDH* in corn (*Zea mays*) can increase its soluble sugar and free amino acid content and thereby improve its cold tolerance [27]. Therefore, *CHDH* (LOC101303901) may be a key gene for low temperature tolerance in wild strawberries.

Compared with the leaves of untreated *F. daltoniana*, 22 starch and sucrose metabolic pathway genes in the leaves of *F. daltoniana* were significantly upregulated under low temperature stress. Cellulose, an intermediate metabolite of sucrose metabolism, can be catalyzed by endoglucanase to produce cellobiose and then catalyzed by β-glucosidase to produce glucose. Compared with the leaves of untreated *F. daltoniana*, after 6 and 12 h of low temperature stress, *EGase* (LOC101313667) increased 6.17- and 8.79-fold, respectively; the glucose content increased 3.63- and 3.83-fold, respectively, and the sucrose content decreased 2.06- and 2.48-fold in the leaves of *F. daltoniana*, respectively.

### The abscisic acid regulatory mechanism of wild strawberry in response to cold stress

Compared with the leaves of untreated *F. daltoniana* and *F. vesca*, the ABA biosynthetic pathway gene *NCED* (LOC101315210) was significantly upregulated in the leaves of *F. daltoniana* and *F. vesca* under low temperature stress, while the ABA degradative gene *CYP707A* (LOC101299092) was significantly downregulated. *NCED* is an important gene that restricts the ABA biosynthetic pathway, and its expression is positively correlated with ABA content in plants [28]. Okamoto et al. found that ABA degradation was reduced in *Arabidopsis CYP707A* mutants [29]. Therefore, *NCED* (LOC101315210) and *CYP707A* (LOC101299092) may play important roles in the accumulation of ABA in wild strawberry leaves under low temperature stress. ABA can phosphorylate SnRK2 by binding to PP2C and inhibiting it, thereby activating the enzymatic activity of SnRK2 [30]. Afterwards, SnRK2 phosphorylates ABF transcription factors and regulates the expression of ABA-induced genes [31]. Previous research has shown that *ABF2* plays an important role in glucose signal transduction and that overexpression can increase the sensitivity of plants to glucose and ABA, thereby enhancing plant resistance [32]. Additionally, the overexpression of *ABF3* and *ABF4* can improve plant cold tolerance and ABA sensitivity [33]. Therefore, compared with the leaves of untreated *F. daltoniana* and *F. vesca*, *ABF2* (LOC101302803) was significantly upregulated in the leaves of *F. daltoniana* and downregulated in the leaves of *F. vesca* under low temperature stress, which may indicate that glucose signal transduction functions at a higher level in *F. daltoniana* under low temperature stress. After low temperature stress, *ABF4* (LOC101291997) was significantly downregulated in *F. vesca* leaves, which reduced its sensitivity to ABA. The difference in the trends of expression of *ABF2* (LOC101302803) and *ABF4* (LOC101291997) in the leaves of *F. daltoniana* and *F. vesca* under low temperature stress could explain the stronger cold tolerance of *F. daltoniana*.

The transcription factors were predicted for the genes of the darkslateblue module that were closely related to REC and ABA content, and thus, nine differentially expressed transcription factors were found, including MJB21.7, GBF1, ERF9, PCL1, RGL1, LBD1, TT2, NF-YB2, and NF-YB11. Studies have shown that NF-YB2, RGL1, ERF9, and GBF1 play important roles in plant resistance mechanisms [34–37]. Sato et al. found that the overexpression of NF-YB2 can significantly enhance drought tolerance by regulating the expression of genes that respond to drought stress, and it is likely to play a role in the ABA-dependent pathway [34]. Achard et al. found that RGL1 can reduce the accumulation of ROS in plants under stress, thereby delaying cell death and enhancing plant tolerance [35]. Dong et al. found that the level of expression of citrus CitERF9 was induced by stress and ABA [37]. ABI5 is an important transcription factor in the ABA-dependent pathway, which can enhance plant stress resistance by regulating the accumulation of osmotic regulators and eliminating ROS [38]. Sun et al. found that the overexpression of *TaGBF1* positively regulates the expression of *ABI5* and increases the salinity and ABA sensitivity of wheat (*Triticum aestivum*) and *Arabidopsis* [36]. Therefore, *NF-YB2* (LOC101295885), *RGL1* (LOC101302310), *ERF9* (LOC101314350), and *GBF1* (LOC101307640) are likely to play important roles in the cold resistance mechanisms of wild strawberries. In particular, *GBF1* (LOC101307640) may regulate the accumulation of glucose and fructose in wild strawberry leaves and the elimination of ROS under low temperature stress.

A correlation analysis revealed that the expression levels of nine differentially expressed transcription factors significantly are correlated with the expression levels of 17 differentially expressed genes in the starch and sucrose metabolic pathways (*P* < 0.05, *R* > 0.8). Expression levels of eight of these differentially expressed transcription factors (all except *ERF9*) were significantly correlated with the expression levels of 10 differentially expressed genes in the amino acid metabolic pathways. This indicates that the differentially expressed transcription factors of the darkslateblue module that were detected are likely to play important roles in the ABA-regulated resistance mechanism of wild strawberry under low temperature stress, particularly for the expression of genes in the starch and sucrose metabolic and amino acid metabolic pathways and the elimination of ROS.

Thus, low temperature stress reduced the activity of POD in the leaves of *F. daltoniana* and *F. vesca*, which is likely to cause a large accumulation of ROS, resulting in a significant increase in MDA content and cell membrane damage (a significant increase in REC), thereby affecting the function of wild strawberry cells. Under low temperature stress, wild strawberry may increase the accumulation of ABA by upregulating the expression of *NCED* (LOC101315210) and downregulating the expression of *CYP707A* (LOC101299092). Under low temperature stress, the ABA content in the leaves of *F. daltoniana* (strong cold tolerance) is significantly higher than that of *F. vesca* (weak cold tolerance) leaves, which could be the root cause of its stronger cold tolerance.

Under low temperature stress, wild strawberry may regulate the expression of transcription factors, such as *NF-YB2* (LOC101295885), *RGL1* (LOC101302310), *ERF9* (LOC101314350), and *GBF1* (LOC101307640), and other transcription factors through ABA to regulate the metabolic pathway genes of sugar-producing amino acids and sugar-producing and ketogenic amino acids to provide more substrates for the biosynthesis of carbohydrates and regulate the level of expression of genes in the starch and sucrose metabolic pathways. These activities significantly increase the glucose and fructose content and the activity of ROS scavenging enzymes, including POD, to eliminate ROS. The primary osmotic adjustment substances that accumulated in wild strawberry leaves under low temperature stress are glucose and fructose, which may play important roles in its mechanism of cold tolerance. The levels of expression of *CHDH* (LOC101303901) and *EGase* (LOC101313667) may play key roles in the accumulation of glucose and fructose in strawberry under low temperature stress. *GBF1* (LOC101307640) may regulate the levels of expression of *CHDH* (LOC101303901) and *EGase* (LOC101313667) under low temperature stress. In addition, compared with the leaves of untreated *F. daltoniana* and *F. vesca*, *ABF2* (LOC101302803) was significantly upregulated in the leaves of *F. daltoniana* and significantly downregulated in those of *F. vesca* under low temperature stress, which suggests that glucose signal transduction plays a greater role in *F. daltoniana* under low temperature stress. Compared with the leaves of untreated *F. vesca*, the level of expression of *ABF4* (LOC101291997) was significantly downregulated in *F. vesca* leaves under low temperature stress, which may reduce its sensitivity to ABA. Thus, this may be one of the reasons for the weak cold tolerance of this species.

## Materials and methods

### Plant materials

Five diploid *Fragaria* species, including *F. nilgerrensis*, *F. vesca*, *F. nubicola*, *F. daltoniana*, and *F. pentaphylla,* were cultivated in a greenhouse at Taizhou University, Taizhou, China. On November 1st, 2020, we selected 20 stolons with five nodes from each species and transplanted them into plastic pots with a diameter of 7.5 mm and filled with a mixture of soil, bark, vermiculite, and sand with a ratio of 2:1:1:2 (*v*:*v*:*v*:*v*). The temperature was maintained at 20/14 °C (day/night), and the relative humidity was 75% [16]. After 1 week of cultivation, the stolons were cut from a single plant. Eight-week-old seedlings were used in the next experiment.

### Cold tolerance evaluation

The cold resistance of the five *Fragaria* species was determined as described by Xue et al. [39]. In brief, leaf discs with a diameter of 6 mm that lacked veins were generated using a hole puncher, washed with distilled water, dried with filter paper, placed on 1 cm thick wet sand prepared in a Petri dish, and then placed at 4 °C for 12 h. The leaves were treated at temperatures of 0 °C, − 4 °C, − 8 °C, − 12 °C, − 16 °C, − 20 °C for 12 h, and the temperature was reduced at a rate of 1 °C/h. The frozen leaves were thawed for 12 h at 4 °C and placed in a centrifuge tube. A volume of 6 mL of deionized water was added to keep the leaves submerged. The samples were then placed under vacuum for 30 min and shaken for 3 h. The electrical conductivity (R_1_) at room temperature was then measured using a DDSJ-308A conductivity meter (Shanghai INESA Scientific Instrument Co., Ltd., Shanghai, China). Afterwards, the solution was autoclaved for 20 min at 122 °C to completely dissolve the plant cell wall and measure the electrical conductivity (*R*_2_). Each treatment consisted of three biological replicates and three technical replicates.

1 $$\mathrm{REC}={\mathrm{R}}_1/{\mathrm{R}}_2\times 100\%$$

2 $$\mathrm{y}=\mathrm{K}/\left(1+{\mathrm{ae}}^{\hbox{-} \mathrm{bx}}\right)$$

3 $$\ln \left[\left(\mathrm{K}\hbox{-}\mathrm{y}\right)/\mathrm{y}\right]=\ln\;\mathrm{a}\hbox{-} \mathrm{bx}$$

4 $${\mathrm{LT}}_{50}=\hbox{-}\left(\ln\mathrm{a}\right)/\mathrm{b}$$

Here, *y* is the relative electrical conductivity (REC); *x* is the treatment temperature; *K* is the maximum limit value of *y*, and *a* and *b* are the parameters of Eq. 4. LT_50_ is the temperature at which 50% of the population died [39].

### Experimental design

Based on the ability of five *Fragaria* species to tolerate cold (Table S1), *F. nilgerrensis* was selected as the cold-tolerant species, while *F. vesca* was selected as the cold-sensitive species. Before the low temperature treatment (0 h), the central lobules of *F. nilgerrensis* and *F. vesca* were collected as the control group for subsequent experiments. *F. nilgerrensis* and *F. vesca* were then placed in an illuminated artificial climate chamber at − 5 °C, and the samples were collected after 6 h and 12 h of low temperature treatment. The sample collection method was the same as that of the control group. The samples were divided into two groups. One group was immediately used to assay the activity of peroxidase (POD) and determine the relative electrical conductivity (REC) and malondialdehyde (MDA) content, while the other group was immediately frozen in liquid nitrogen and stored at − 80 °C to determine the ABA content and perform transcriptome sequencing and metabolomic identification.

### Physiological index measurement

#### Assay for peroxidase activity

A volume of 5 mL of 50 mM Na phosphate buffer (pH = 7.8) was added to 0.5 g of leaves, ground into a homogenate and centrifuged for 20 min at 10,000 rpm at 4 °C. The supernatant was transferred to a new tube. The POD activity was determined by the nitrogen blue tetrazolium (NBT) photochemical reduction method [40]. The reaction system included 50 mM phosphate buffer (pH 7.8), 13 mM methionine, 75 mM NBT, 0.1 mM EDTA, 50 mM Na_2_CO_3_, and 100 μL of crude enzyme solution. The reaction solution was irradiated with two 20 W fluorescent tubes for 15 min. The reaction solution was subjected to colorimetric analysis at 560 nm, and a reduction of 50% in the absorbance of NBT was considered to be one unit of activity (U).

#### Determination of the malondialdehyde content

A volume of 10 mL of 10% trichloroacetic acid (TCA) and 1 g of fresh leaves were added to a mortar and ground. The homogenate was centrifuged at 10,000 rpm at 4 °C for 20 min. The reaction mixture that contained 2 mL of extract and 2 mL of 0.6% thiobarbituric acid (TBA) was placed in a water bath at 95 °C for 30 min, quickly cooled on ice, and then centrifuged again at 10,000 rpm for 20 min. The absorbance of the supernatant was measured at 532, 600, and 450 nm with a T6 New Century UV-VIS spectrophotometer (Beijing Persee General Instrument Co. Ltd., Beijing, China), and A_532_, A_600_, and A_450_ were obtained, respectively [41].

5 $$\mathrm{MDA}\;\mathrm{content}=6.45\times \left({\mathrm{A}}_{532}\hbox{-} {\mathrm{A}}_{600}\right)\hbox{-} 0.56\times {\mathrm{A}}_{450}$$

#### Determination of relative electrical conductivity

The REC was the same as that determined in section 4.2.

#### Determination of abscisic acid content

The frozen leaves were ground to powder with an MM 400 grinder (Retsch Technology GmbH, Haan, Germany). A total of 50 mg of powder was added with an appropriate amount of internal standard and extracted with 1 mL of methanol:water:formic acid (15:4:1, *v*:*v*:*v*). After concentration, it was reconstituted with 100 μL of 80% methanol and filtered through a 0.22-μm membrane. Ultra performance liquid chromatography (UPLC) (ExionLC™ AD, https://sciex.com.cn/) and tandem mass spectrometry (QTRAP® 6500+, https://sciex.com.cn/) were used to determine the concentrations of hormones as described by Chen et al. [42].

### Transcriptomic analysis

#### Total RNA extraction, library construction, and transcriptome sequencing

The total RNA was extracted using a TRIzol kit (B511311; Sangon, Shanghai, China) according to the manufacturer’s instructions. The purity and concentration of RNA were detected by spectrophotometry (IMPLEN, Westlake Village, CA, USA) and a Qubit® RNA Analysis Kit (Life Technologies Corporation, Carlsbad, CA, USA). The integrity of RNA was evaluated by an RNA Nano 6000 Analysis Kit on an Agilent 2100 Bioanalyzer (Agilent Technologies, Santa Clara, CA, USA). A VAHTSTM mRNA-seq V2 Library Preparation Kit (Illumina, San Diego, CA, USA) was used to generate the sequencing libraries. A HiSeq X Ten sequencer (Illumina) was used for paired-end sequencing of the library. The extraction of total RNA and sequencing was completed by Shenggong Bioengineering Co., Ltd. (Shanghai, China).

#### Sequence alignment

First, we used FastQC (version 0.11.2) to remove low-quality sequencing reads in the original data, such as adaptor sequences and low-quality bases. After that, we used HISAT2 (version 2.1.0) software to compare the filtered data to the genome of the strawberry model species *F. vesca* under the default parameters of the software (https://www.ncbi.nlm.nih.gov/assembly/GCA_000184155.1).

#### Identification and analysis of differentially expressed genes

The differentially expressed genes (DEGs) were screened using DESeq software (version 1.12.4), according to the following criteria: *q*-value < 0.001, |Fold Change| > 2. The Gene Ontology (GO) and Kyoto Encyclopedia of Genes and Genomes (KEGG) enrichment analyses of DEGs were performed using the topGO (version 2.24.0) and clusterProfiler (version 3.0.5) packages for the R statistical computing environment, respectively [43–46].

#### Transcription factor prediction

The sequence file was uploaded to the PlantTFDB website to predict the transcription factors (http://planttfdb.cbi.pku.edu.cn/index.php) [47].

#### Weighted gene co-expression network analysis (WGCNA)

The WGCNA software package in R (version 3.6.3) was used for the WGCNA analysis of mRNAs and plant physiological indicators as described by Lu et al. [16]. After filtering out genes with a low level of expression (fragments per kilobase of transcript per million mapped fragments [FPKM] < 0.5), the threshold parameter (β value) was determined and established by the soft threshold method. Then, the correlation coefficient between expression levels of genes was converted by the adjacency function to form an adjacency matrix, which was then converted into a topological matrix based on the TOM similarity algorithm. The degree of difference between expression levels of the genes was calculated based on this. The minimum number of genes in the module was set to 30, and the hybrid dynamic shearing tree method was used to generate a dendritic clustering graph. After the module was constructed, the correlation analysis between the module and the phenotype was performed, and the GO and KEGG enrichment analyses of the target module gene were conducted.

#### Correlation analysis

The Pearson correlation coefficients between the DEGs and transcription factors, the log_2_ conversion of expression levels of DEGs and of accumulation levels of metabolites were calculated using the Cor function in R (version 3.5.1). CytoScape (version 3.6.1) software (Shannon, 2003) was used to visualize the data for which *P* < 0.05 and *R* > 0.8 [48].

#### qRT-PCR experiment

To verify the mRNA expression pattern identified by RNA-Seq, six DEGs were screened, and their levels of expression were verified by real-time fluorescent quantitative reverse transcription PCR (qRT-PCR) on a C1000 Touch fluorescent quantitative PCR instrument (Bio-Rad Co. Ltd., Hercules, CA, USA). The primers (Table S2) were designed using Primer Premier 5.0 and synthesized by Sangon Biotech Co., Ltd. (Shanghai, China). The strawberry actin gene was used as an internal reference gene, and the relative expression of the gene was calculated by the 2^-ΔΔCT^ method. All qRT-PCR experiments contained three sets of biological replicates and three sets of technical replicates.

### Metabolomic analysis

Frozen leaves were ground at 30 Hz with an MM 400 grinder (Retsch Technology GmbH, Haan, Germany) for 1.5 min. A total of 100 mg of powder was dissolved in a 1.0-mL solution of methanol that contained 0.1 mg/L lidocaine (internal standard) and 70% methanol. The samples were incubated overnight at 4 °C, during which they were shaken three times to ensure a more thorough extraction. After extraction, the samples were centrifuged for 10 min at 4 °C at 10,000 rpm, and the supernatants were filtered through 0.22-μm membranes.

As described by Wei et al. [49], the metabolites were identified and quantified by UPLC and tandem mass spectrometry (MS/MS, Applied Biosystems 4500 QTRAP, http://www.appliedbiosystems.com.cn/). The conditions for the liquid phase are described as follows: the chromatographic column was an Agilent SB-C18 (1.8 μm, 2.1 mm × 100 mm); the mobile phase was ultrapure water that contained 0.1% formic acid as phase A and acetonitrile that contained 0.1% formic acid as phase B; the elution gradient was set to 5% of phase B at 0 min, the ratio of phase B increased linearly to 95% within 9 min, which was maintained for 1 min, at 10–11 min, the ratio of phase B was reduced to 5%, and the ratio was balanced to 14 min.

The operating parameters of the ESI source were set as follows: ion source, turbo spray; source temperature, 550 °C; ion spray voltage, 5500 V; ion source gas I, gas II, and curtain gas pressures, 55, 60, and 25 psi respectively; collision-induced ionization parameter, high. In the QQQ and LIT modes, 10 and 100 μmol/L polypropylene glycol solutions were used to tune the instrument and calibrate the mass, respectively. A QQQ scan selected the multiple reaction monitoring (MRM) mode, and the collision gas (nitrogen) was set to medium.

The metabolites were analyzed qualitatively based on HMDB (http://www.hmdb.ca/), MassBank (http://www.massbank.jp/), and the KNAPSAcK (http://kanaya.naist.jp/KNApSAcK/) database [50]. The characterization of metabolites was based on MRM scans by checking the five parameters DP, CE, RT, Q1, and Q3 of the detected sample substances with database data [49]. The quantification of metabolites involved first integrating and correcting the mass spectral peak areas of all substances through MRM and then integrating and correcting the mass spectral peak areas of the same metabolite in separate samples [51]. Metabolites with variable importance for projection (VIP) ≥ 1.0 and fold change ≥2 or fold change ≤0.5 were considered significantly different.

### Statistical analysis

One-way analysis of variance (ANOVA) was used to test the differences among the means of variables and Duncan’s multiple range test was used for post hoc pairwise comparison of different treatments. All the statistical analyses were performed using SAS software (version 9.4; SAS Institute, Cary, NC, USA).

## Conclusions

In this study, an integrated analysis of transcriptome, metabolome, and physiological index data revealed an ABA regulation mechanism of two wild *Fragaria* species in response to cold stress. Under low temperature stress, wild strawberry may increase the accumulation of ABA by regulating the expression of ABA biosynthetic and metabolic pathway genes. After that, ABA may regulate the level of expression of amino acid metabolism and sucrose metabolism pathway genes by regulating GBF1 and other transcription factors to significantly increase the content of glucose and fructose and increase the activity of ROS scavenging enzymes, including POD, thereby reducing the damage caused by low temperature stress. The expression of ABF2 may be related to the glucose signal transduction ability of wild strawberry under low temperature stress. Under low temperature stress, the differences in the accumulation of ABA and the trends of expression of ABF2 and ABF4 in different species of wild strawberries may be the primary reason for their differences in cold tolerance.

## Supplementary Information

**Additional file 1: Fig. S1.** Determination of the soft threshold.

**Additional file 2: Fig. S2.** GO enrichment analysis of ABA and REC related modular genes. ABA, abscisic acid; GO, gene ontology; REC, relative electrical conductivity.

**Additional file 3: Fig. S3.** KEGG enrichment analysis of modular genes. (a) Modular genes related to ABA and REC. (b) Modular genes related to POD and MDA. ABA, abscisic acid; KEGG, Kyoto Encyclopedia of Genes and Genomes; MDA, malondialdehyde; POD, peroxidase; REC, relative electrical conductivity.

**Additional file 4: Fig. S4.** KEGG functional annotation of ABA and REC related module genes. ABA, abscisic acid; KEGG, Kyoto Encyclopedia of Genes and Genomes; REC, relative electrical conductivity.

**Additional file 5: Table S1.** The cold tolerance ability of five *Fragaria* species.

**Additional file 6: Table S2.** Primers used for qRT-PCR.

**Additional file 7: Table S3.** The VIP and fold change value of the contents amino acids and intermediate metabolites in their metabolic pathways in leaves of *F. vesca* and *F. daltoniana* under cold stress. VIP, variable importance in projection

**Additional file 8: Table S4.** The VIP and fold change value of the sugar content in leaves of *F. vesca* and *F. daltoniana* under cold stress. VIP, variable importance in projection.

## Data Availability

All raw data have been submitted to the National Center for Biotechnology Information (NCBI) database (under the BioProject accession number PRJNA803876).
