# Supplementary figures and images for: The mechanism of abscisic acid regulation of wild Fragaria species in response to cold stress
Source: BMC Genomics. 2022 Sep 26;23:670. doi: 10.1186/s12864-022-08889-8 (PMC9513977; doi:10.1186/s12864-022-08889-8)

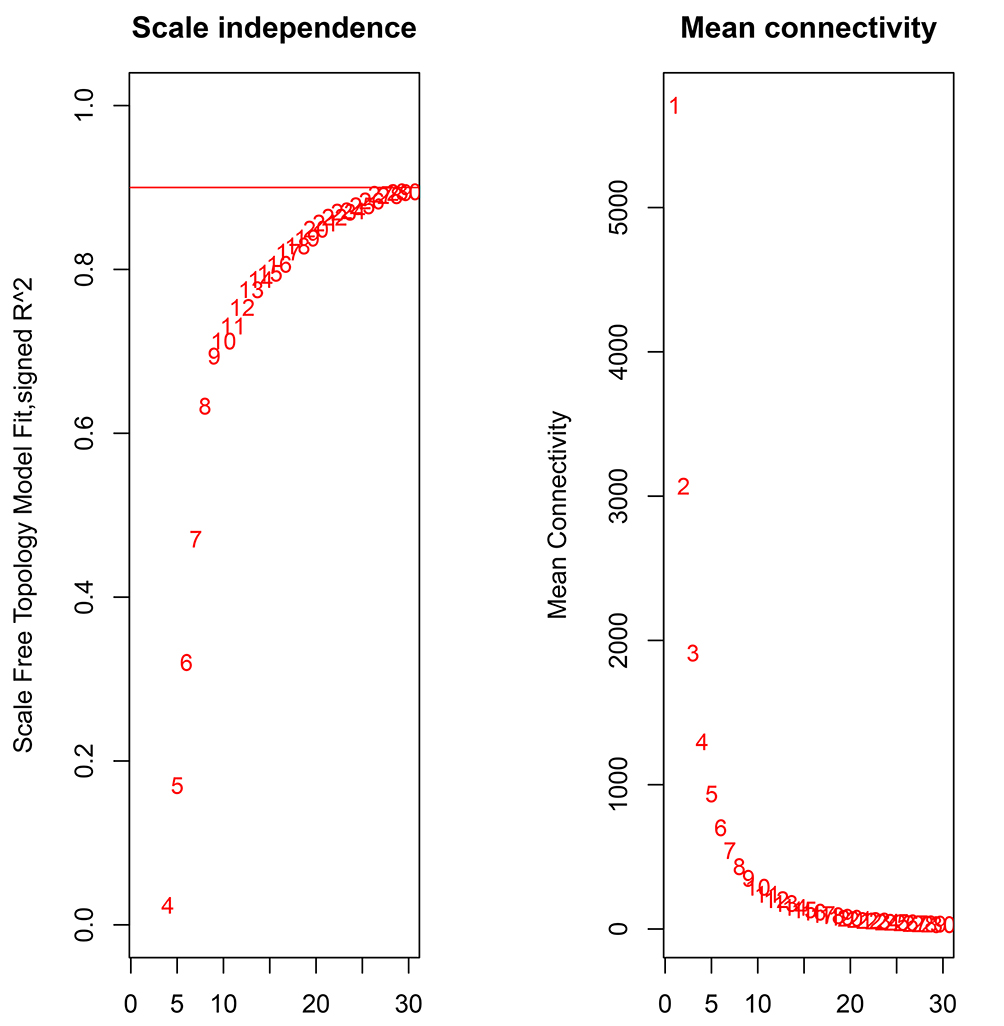

Supplement: Supplementary file 1 — Additional file 1: Fig. S1. Determination of the soft threshold. [file 12864_2022_8889_MOESM1_ESM.jpg]

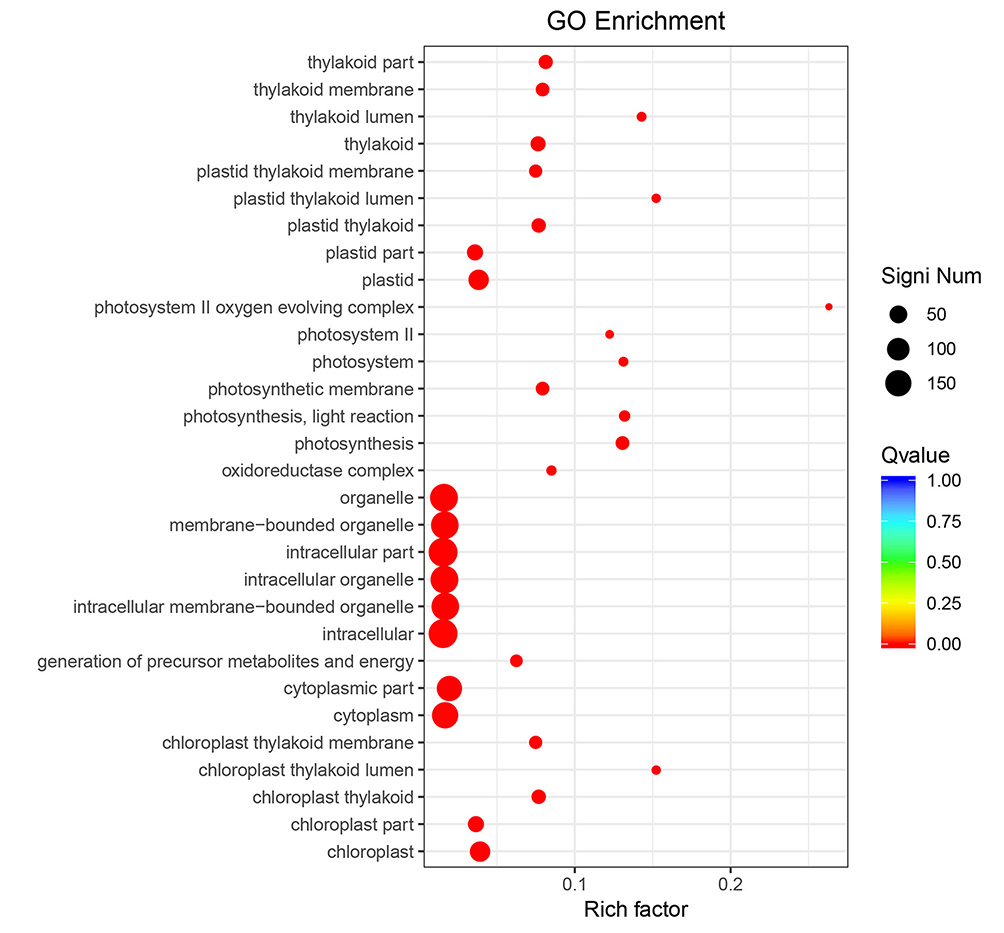

Supplement: Supplementary file 2 — Additional file 2: Fig. S2. GO enrichment analysis of ABA and REC related modular genes. ABA, abscisic acid; GO, gene ontology; REC, relative electrical conductivity. [file 12864_2022_8889_MOESM2_ESM.jpg]

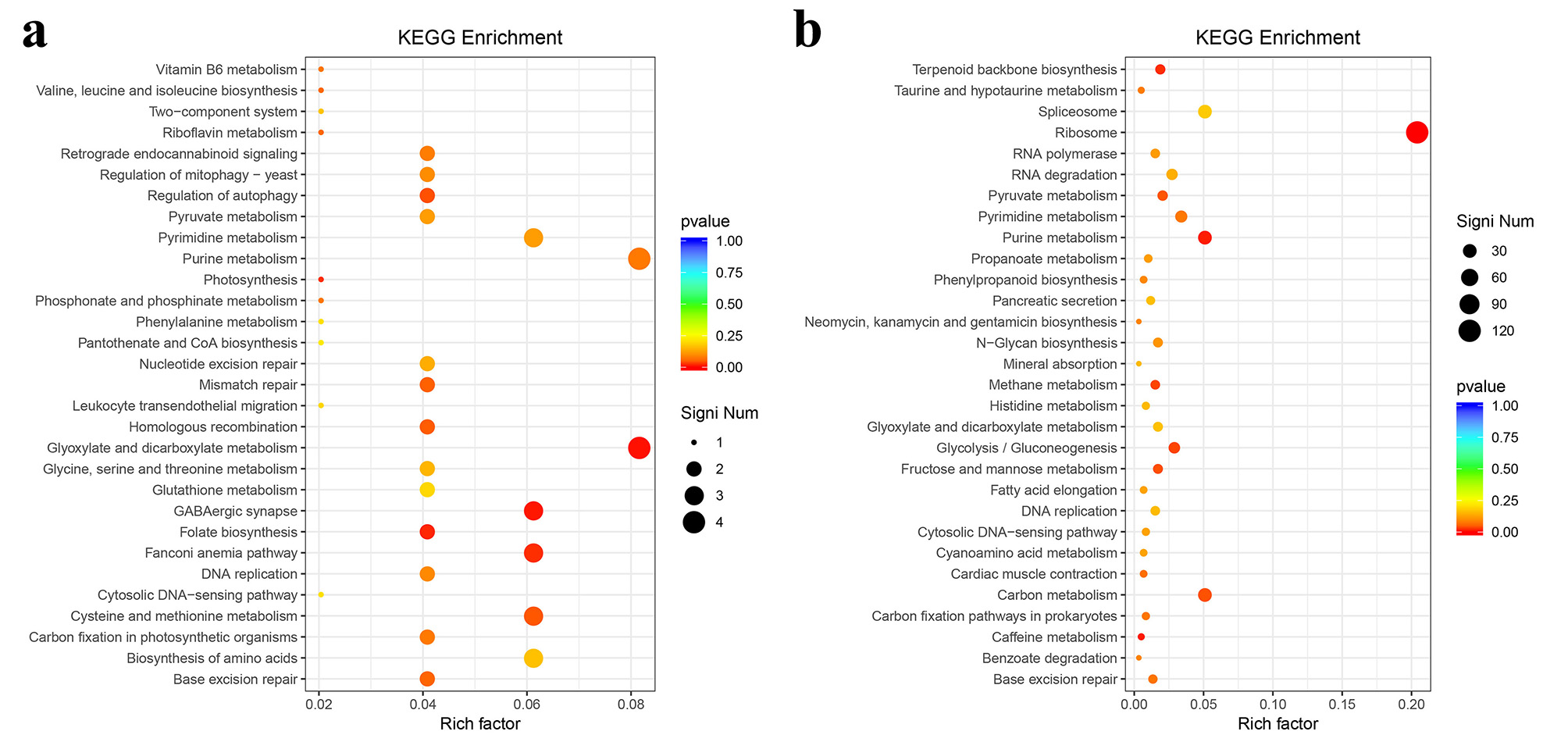

Supplement: Supplementary file 3 — Additional file 3: Fig. S3. KEGG enrichment analysis of modular genes. (a) Modular genes related to ABA and REC. (b) Modular genes related to POD and MDA. ABA, abscisic acid; KEGG, Kyoto Encyclopedia of Genes and Genomes; MDA, malondialdehyde; POD, peroxidase; REC, relative electrical conductivity. [file 12864_2022_8889_MOESM3_ESM.jpg]

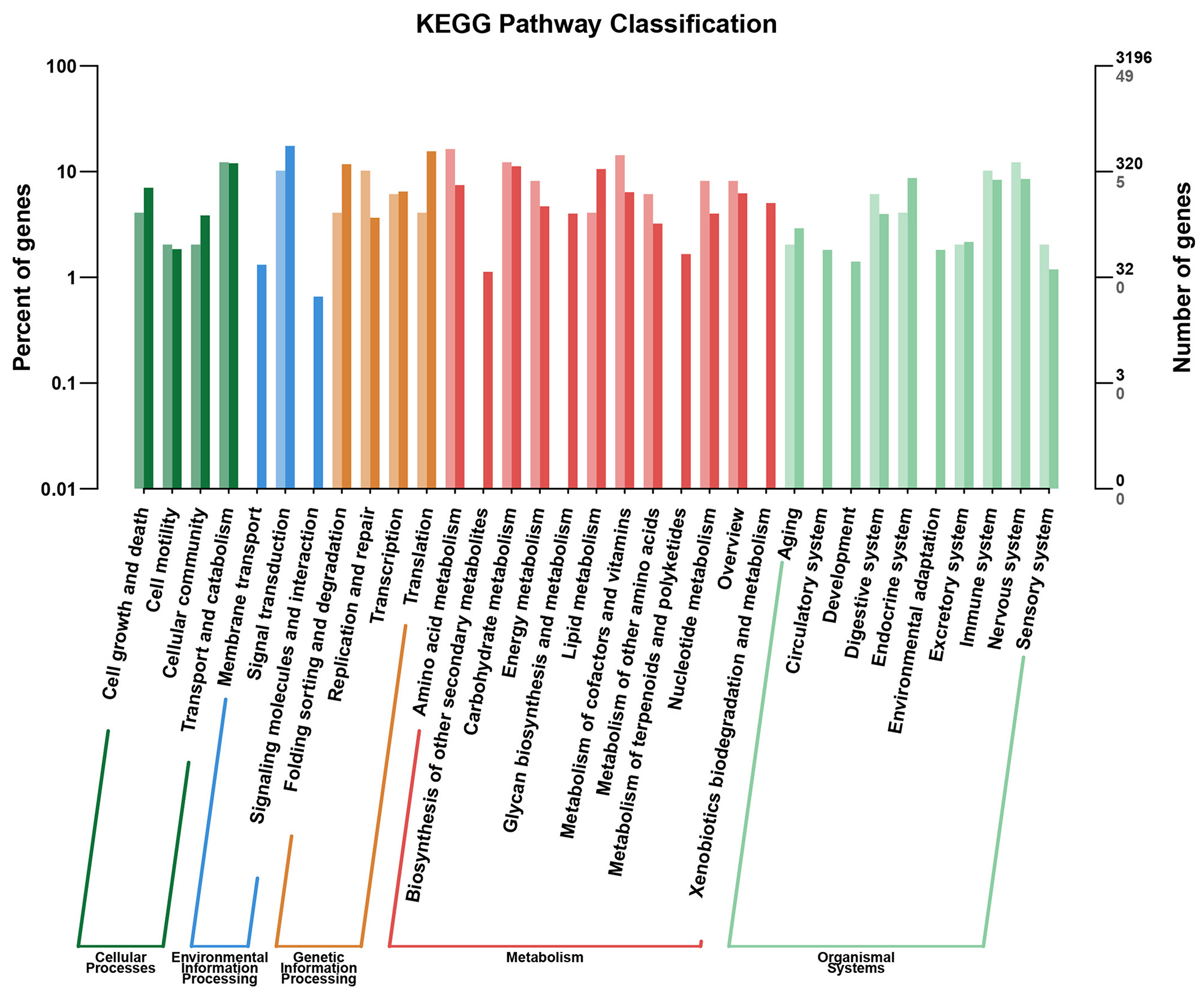

Supplement: Supplementary file 4 — Additional file 4: Fig. S4. KEGG functional annotation of ABA and REC related module genes. ABA, abscisic acid; KEGG, Kyoto Encyclopedia of Genes and Genomes; REC, relative electrical conductivity. [file 12864_2022_8889_MOESM4_ESM.jpg]
