# Supplementary material for: The mechanism of abscisic acid regulation of wild Fragaria species in response to cold stress
Source: BMC Genomics. 2022 Sep 26;23:670. doi: 10.1186/s12864-022-08889-8 (PMC9513977; doi:10.1186/s12864-022-08889-8)
Supplement: Supplementary file 5 — Additional file 5: Table S1. The cold tolerance ability of five Fragaria species. [file 12864_2022_8889_MOESM5_ESM.docx]

Table S1 The cold tolerance ability of five *Fragaria* species

| Varieties | Logistic equation | *R*^2^ | LT_50_（^o^C） |
| --- | --- | --- | --- |
| *Fragaria nubicola* | Y=77.1005/(1+6.0720*e^0.1729*x^) | 0.8632 | -10.4350 |
| *Fragaria vesca* | Y=75.1649/(1+2.7649*e^0.1143*x^) | 0.8589 | -8.8952 |
| *Fragaria daltoniana* | Y=74.8474/(1+6.8377*e^0.1726*x^) | 0.8780 | -11.1389 |
| *Fragaria pentaphylla* | Y=78.2584/(1+3.4552*e^0.1335*x^) | 0.9594 | -9.2887 |
| *Fragaria nilgerrensis* | Y=78.4091/(1+7.9626*e^0.1981*x^) | 0.8978 | -10.4711 |

LT_50_: semi-lethal temperature
