# Supplementary material for: The mechanism of abscisic acid regulation of wild Fragaria species in response to cold stress
Source: BMC Genomics. 2022 Sep 26;23:670. doi: 10.1186/s12864-022-08889-8 (PMC9513977; doi:10.1186/s12864-022-08889-8)
Supplement: Supplementary file 6 — Additional file 6: Table S2. Primers used for qRT-PCR. [file 12864_2022_8889_MOESM6_ESM.docx]

Table S2 Primers used for qRT-PCR

| Gene ID | Forward | Reverse |
| --- | --- | --- |
| LOC101314350 | ACAACGGCGGGAATAGCAACAG | CAACGGCGAGGACTCAACCATC |
| LOC101300227 | TGGTTAGTCCTTGTGCTGCTTGC | CTATGGTGAACTTGGCTGGCTCAG |
| LOC101296688 | GCATGGACAGCTATGGAGGACAG | AGCCTGCAACTCTTTCCACATCTC |
| LOC101295885 | GAGGAGGAGCAGGAGCAGGAG | CCAATCCCAACCCCACCAACAC |
| LOC101313667 | GGTGGGTTGATGAAGGGTGAGTTG | GGACATAAATGGTGCCTGGGTGAG |
| LOC105349241 | TGAGTTTCTTGGACAAGCCGAGTG | GCCAGGAGCAACTTCGTCGTC |
| Actin | TTCACGAGACCACCTATAACTC | GCTCATCCTATCAGCGATT |
