# Supplementary material for: The mechanism of abscisic acid regulation of wild Fragaria species in response to cold stress
Source: BMC Genomics. 2022 Sep 26;23:670. doi: 10.1186/s12864-022-08889-8 (PMC9513977; doi:10.1186/s12864-022-08889-8)
Supplement: Supplementary file 7 — Additional file 7: Table S3. The VIP and fold change value of the contents amino acids and intermediate metabolites in their metabolic pathways in leaves of F. vesca and F. daltoniana under cold stress. VIP, variable importance in projection [file 12864_2022_8889_MOESM7_ESM.docx]

Table S3. The VIP and fold change value of the contents amino acids and intermediate metabolites in their metabolic pathways in leaves of *F. vesca* and *F. daltoniana* under cold stress. VIP, variable importance in projection.

| Metabolite | Fd_CK vs. Fd_LT_6h | | Fd_CK vs. Fd_LT_12h | | Fv_CK vs. Fv_LT_6h | | Fv_CK vs. Fv_LT_12h | |
| --- | --- | --- | --- | --- | --- | --- | --- | --- |
|  | VIP | Fold  Change | VIP | Fold  Change | VIP | Fold  Change | VIP | Fold  Change |
| L-Arginine | 1.01 | 0.42 | 1.09 | 0.44 | 0.04 | 1.01 | 0.21 | 1.14 |
| L-Asparagine | 1.39 | 0.49 | 1.29 | 0.41 | 0.05 | 1.02 | 0.73 | 0.78 |
| L-Aspartic Acid | 1.42 | 0.28 | 1.38 | 0.19 | 1.51 | 0.49 | 1.52 | 0.36 |
| L-Citrulline | 1.29 | 0.33 | 1.17 | 0.45 | 1.11 | 1.95 | 0.86 | 0.69 |
| L-Cysteine | 0.31 | 0.97 | 0.86 | 0.69 | 0.52 | 0.93 | 0.84 | 0.80 |
| L-Cystine | 1.35 | 3.57 | 1.35 | 2.71 | 1.51 | 3.60 | 1.53 | 3.01 |
| L-Glutamic acid | 1.35 | 0.26 | 1.40 | 0.14 | 1.42 | 0.54 | 1.46 | 0.41 |
| L-Histidine | 0.37 | 0.74 | 0.64 | 0.59 | 0.02 | 1.00 | 1.02 | 1.20 |
| L-Homocitrulline | 0.26 | 1.11 | 1.18 | 1.38 | 0.37 | 0.85 | 0.71 | 1.45 |
| L-Homocystine | 0.84 | 1.34 | 1.20 | 1.51 | 1.49 | 1.34 | 1.17 | 1.77 |
| L-Homomethionine | 1.32 | 0.44 | 1.35 | 0.37 | 0.66 | 0.86 | 0.74 | 0.74 |
| L-Isoleucine | 1.04 | 1.24 | 1.11 | 1.29 | 0.79 | 0.90 | 1.03 | 0.87 |
| L-Leucine | 1.00 | 1.25 | 1.13 | 1.35 | 0.95 | 0.86 | 0.97 | 0.86 |
| L-Lysine | 0.62 | 0.84 | 0.64 | 0.85 | 0.76 | 1.16 | 0.69 | 1.09 |
| L-Methionine | 1.08 | 1.73 | 1.18 | 1.88 | 0.82 | 1.12 | 0.27 | 1.04 |
| L-Norleucine | 0.94 | 1.21 | 1.18 | 1.37 | 0.71 | 0.91 | 0.79 | 0.88 |
| L-Ornithine | 1.36 | 0.50 | 1.20 | 0.45 | 0.70 | 1.31 | 0.39 | 0.98 |
| L-Phenylalanine | 1.14 | 1.22 | 1.18 | 1.44 | 1.25 | 1.25 | 1.16 | 1.34 |
| L-Proline | 0.09 | 0.99 | 0.20 | 1.04 | 1.25 | 1.44 | 0.15 | 1.03 |
| L-Serine | 1.43 | 0.40 | 1.24 | 0.62 | 0.11 | 0.94 | 0.70 | 0.74 |
| L-Threonine | 1.23 | 0.63 | 1.33 | 0.51 | 0.46 | 0.78 | 0.58 | 0.77 |
| L-Tryptophan | 0.66 | 1.11 | 0.40 | 1.10 | 1.47 | 1.26 | 1.47 | 1.96 |
| L-Tyrosine | 1.19 | 0.78 | 0.31 | 1.04 | 1.66 | 1.61 | 1.35 | 1.73 |
| L-Valine | 0.02 | 1.00 | 0.38 | 0.95 | 0.45 | 1.05 | 1.18 | 0.83 |
| Dimethylglycine | 1.45 | 54.70 | 1.40 | 53.50 | 1.66 | 7.87 | 1.58 | 7.28 |

^*^Fv: *F. vesca*; Fd: *F. daltoniana*; CK: Control group；LT: Low temperature.
