# Supplementary material for: The mechanism of abscisic acid regulation of wild Fragaria species in response to cold stress
Source: BMC Genomics. 2022 Sep 26;23:670. doi: 10.1186/s12864-022-08889-8 (PMC9513977; doi:10.1186/s12864-022-08889-8)
Supplement: Supplementary file 8 — Additional file 8: Table S4. The VIP and fold change value of the sugar content in leaves of F. vesca and F. daltoniana under cold stress. VIP, variable importance in projection. [file 12864_2022_8889_MOESM8_ESM.docx]

Table S4. The VIP and fold change value of the sugar content in leaves of *F. vesca* and *F. daltoniana* under cold stress. VIP, variable importance in projection.

| Metabolite | Fd_CK vs. Fd_LT_6h | | Fd_CK vs. Fd_LT_12h | | Fv_CK vs. Fv_LT_6h | | Fv_CK vs. Fv_LT_12h | |
| --- | --- | --- | --- | --- | --- | --- | --- | --- |
|  | VIP | Fold  Change | VIP | Fold  Change | VIP | Fold  Change | VIP | Fold  Change |
| D-(-)-Threose | 0.94 | 0.60 | 1.14 | 0.40 | 1.25 | 1.57 | 1.30 | 1.54 |
| D-Arabinose | 0.35 | 1.37 | 1.17 | 1.99 | 1.34 | 1.67 | 1.31 | 2.65 |
| D-Fructose | 1.45 | 3.88 | 1.40 | 4.07 | 1.66 | 2.84 | 1.55 | 4.28 |
| D-Glucose | 1.45 | 3.63 | 1.40 | 3.83 | 1.67 | 2.86 | 1.55 | 4.20 |
| D-Lactulose | 1.36 | 0.46 | 1.32 | 0.50 | 0.59 | 0.72 | 0.11 | 1.03 |
| D-Maltose | 1.24 | 0.68 | 1.38 | 0.54 | 0.00 | 0.96 | 0.24 | 1.29 |
| D-Panose | 0.43 | 0.67 | 0.99 | 0.00 | 0.41 | 0.86 | 0.54 | 1.37 |
| D-Ribose | 1.34 | 1.38 | 1.11 | 1.22 | 1.26 | 1.73 | 1.38 | 1.89 |
| D-Sucrose | 1.15 | 0.49 | 1.29 | 0.40 | 0.25 | 0.77 | 0.09 | 1.00 |
| D-Trehalose | 1.01 | 0.69 | 0.87 | 0.77 | 0.12 | 0.88 | 0.28 | 1.10 |
| Isomaltulose | 1.37 | 0.56 | 1.37 | 0.59 | 0.18 | 0.88 | 0.00 | 1.06 |
| Lactobiose | 1.17 | 0.56 | 1.14 | 0.63 | 0.05 | 0.93 | 0.07 | 1.09 |
| Melibiose | 1.38 | 0.56 | 1.38 | 0.59 | 0.09 | 0.94 | 0.15 | 1.12 |
| Raffinose | 1.14 | 0.47 | 1.23 | 0.50 | 0.80 | 0.59 | 0.09 | 0.96 |
| Sedoheptulose | 0.41 | 1.08 | 0.43 | 1.05 | 0.95 | 1.24 | 0.99 | 1.62 |
| Turanose | 1.06 | 0.64 | 0.96 | 0.69 | 0.49 | 1.07 | 1.10 | 0.77 |

*Fv: *F. vesca*; Fd: *F. daltoniana*; CK: Control group；LT: Low temperature.
